# Feasibility of real time integration of high-resolution scar images with invasive electrograms in electro-anatomical mapping system in patients undergoing ventricular tachycardia ablation

**DOI:** 10.1186/1532-429X-15-S1-E94

**Published:** 2013-01-30

**Authors:** Sébastien Roujol, Tamer A Basha, Alex Y Tan, Elad Anter, Alfred E Buxton, Mark E Josephson, Reza Nezafat

**Affiliations:** 1 Medecine, BIDMC / Harvard Medical School, Boston, MA, USA

## Background

Ventricular tachycardia (VT) ablation is generally guided by invasive mapping of the left ventricle (LV) using electro-anatomical voltage mapping (EAM) to identify the VT substrate [1]. Late gadolinium enhancement (LGE) MRI allows excellent visualization of the scar. Heterogeneous area in LGE images has been shown to correlate with the VT substrate in animal models of VT. Retrospective studies in patients have also correlated the LGE signal enhancement to low voltage in EAM maps. However, current clinical EAM platform such as the Carto3 (Biosense Webster) does not allow integration of LGE images for facilitating the VT ablation. In this study, we described a workflow to integrate scar geometry extracted from high-resolution 3D LGE images with EAM.

## Methods

**Figure 1:**
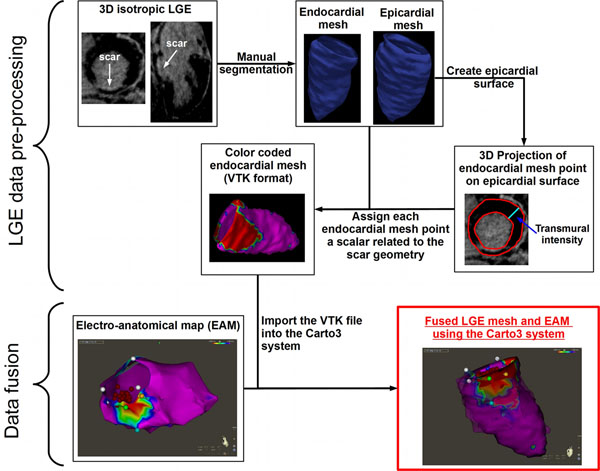
Proposed workflow for integrating 3D high-resolution LGE into clinically available EAM system (Carto3) for VT ablation. Fusion of the imported VTK mesh and EAM is performed using the available landmark registration with 4 landmarks at the base and 1 at the apex within Carto3 system.

The proposed workflow for LGE scar integration into the Carto3 system is described in figure 1. A 3D high-resolution LGE sequence (1.3×1.3×1.3 mm^3^ at 1.5T) [2] is first acquired prior to the VT ablation procedure. Endocardial and epicardial contours are manually drawn on LGE data using an in-house software developed in Matlab (Mathworks, Nattick MA). All remaining LGE data processing steps are performed automatically. Two 3D mesh geometries are generated by interpolation of the drawn contours. A 3D binary volume representing the epicardial surface is also generated from the epicardial mesh. Each endocardial mesh points are projected to the binary epicardial surface by using a rapid minimal distance search to the nearest point on the binary representation of the epicardial surface. The transmural intensity is then extracted for each endocardial mesh point and can be used to measure spatial characteristics of the scar such as the scar thickness or the scar transmurality level. The scar spatial characteristic of each endocardial mesh point is then color coded and the overall endocardial mesh is saved into the VTK format (Kitware Inc). This file is then imported into the Carto3 system before the ablation procedure and is fused with EAM using the landmark registration tool available into the Carto3 system. The feasibility of the proposed workflow is demonstrated in a VT patient undergoing a high resolution LGE exam followed by a VT ablation.

## Results

Using the proposed methodology, a VTK file representing the average transmural intensity of LGE was successfully generated (Figure 1). The LGE data processing was performed in ~20min (manual contour delineation ~20min and all remaining steps ~10s). The resulting VTK file was imported into the Carto3 system before ablation and successfully merged with EAM (Figure 1).

## Conclusions

Real time integration of scar from high-resolution 3D LGE into the Carto3 system is now feasible which allows visualization of LGE scar, EAM voltage map and catheter location in real-time. Further clinical validation is needed to investigate the potential benefit of real time LGE integration for VT ablation guidance.

## Funding

NIH:R01EB008743-01A2
